# In-Car Nocturnal Blue Light Exposure Improves Motorway Driving: A Randomized Controlled Trial

**DOI:** 10.1371/journal.pone.0046750

**Published:** 2012-10-19

**Authors:** Jacques Taillard, Aurore Capelli, Patricia Sagaspe, Anna Anund, Torbjorn Akerstedt, Pierre Philip

**Affiliations:** 1 University of Bordeaux, Sommeil, Attention et Neuropsychiatrie, USR 3413, Bordeaux, France; 2 CNRS, Sommeil, Attention et Neuropsychiatrie, USR 3413, Bordeaux, France; 3 Swedish Road and Transport Research Institute (VTI), Linköping, Sweden; 4 Stress Research Institute, Stockholm University, Stockholm, Sweden; University of Alabama at Birmingham, United States of America

## Abstract

Prolonged wakefulness greatly decreases nocturnal driving performance. The development of in-car countermeasures is a future challenge to prevent sleep-related accidents. The aim of this study is to determine whether continuous exposure to monochromatic light in the short wavelengths (blue light), placed on the dashboard, improves night-time driving performance. In this randomized, double-blind, placebo-controlled, cross-over study, 48 healthy male participants (aged 20–50 years) drove 400 km (250 miles) on motorway during night-time. They randomly and consecutively received either continuous blue light exposure (GOLite, Philips, 468 nm) during driving or 2*200 mg of caffeine or placebo of caffeine before and during the break. Treatments were separated by at least 1 week. The outcomes were number of inappropriate line crossings (ILC) and mean standard deviation of the lateral position (SDLP). Eight participants (17%) complained about dazzle during blue light exposure and were removed from the analysis. Results from the 40 remaining participants (mean age ± SD: 32.9±11.1) showed that countermeasures reduced the number of inappropriate line crossings (ILC) (F(2,91.11) = 6.64; p<0.05). Indeed, ILC were lower with coffee (12.51 [95% CI, 5.86 to 19.66], p = 0.001) and blue light (14.58 [CI, 8.75 to 22.58], p = 0.003) than with placebo (26.42 [CI, 19.90 to 33.71]). Similar results were found for SDLP. Treatments did not modify the quality, quantity and timing of 3 subsequent nocturnal sleep episodes. Despite a lesser tolerance, a non-inferior efficacy of continuous nocturnal blue light exposure compared with caffeine suggests that this in-car countermeasure, used occasionally, could be used to fight nocturnal sleepiness at the wheel in blue light-tolerant drivers, whatever their age. More studies are needed to determine the reproducibility of data and to verify if it can be generalized to women.

**Trial Registration:**

ClinicalTrials.gov NCT01070004

## Introduction

Our society induces behavioural reduction of daily sleep duration and extensive periods of wakefulness or activities in the circadian deep (3–5 a.m.) which are causes of sleepiness-related accidents [1], especially traffic accidents. Owing to conflicts between physiological needs and social [2] or professional activities, countermeasures to fight this sleepiness need to be developed. To date, caffeine and naps have been shown to be effective in real-life driving studies [3], [4] but they have some limitations (differences between individuals in terms of efficiency, limited efficiency duration, side-effects). Caffeine and nap, self-administered countermeasures that involve stopping the car at rest stops, are not adequate because a study has shown that many sleepy drivers (46%) continue to drive [5]. Reasons for not stopping include being far from any possible rest stops, being relatively close to one's destination, or lack of insight into one's state of alertness [5]. When drivers do not stop driving, they use in-car countermeasures (e.g. opening window or turning on the radio) [5] which are insufficient [6], [7]. The development and evaluation of in-car and preventive countermeasures is a major public health issue for the prevention sleepiness-related accidents.

Intrinsically photosensitive retinal ganglion cells that contain melanopsin photopigment known to respond directly to light with a peak spectral sensitivity in the short-wavelength range (460–480 nm blue-light) [8] projects to the suprachiasmatic nucleus (master biological clock) and to the brain area involved in the regulation of arousal [9] by a specific non-visual tract. Continuous exposure to blue light has proved effective on subjective and objective correlates of alertness and performance in the evening [10] and at night [2], [11]. This type of continuous exposure at levels as low as 20 lux could be used as often as required during night-driving as a preventive countermeasure to sleepiness at the wheel and to reduce the risk of accidents in sleepy drivers.

We conducted a randomized, double-blind, placebo-controlled, crossover study to compare the effects of continuous exposure to monochromatic light in the short wavelengths (blue light, 468 nm), placed in the middle of the dashboard, with coffee (2*200 mg of caffeine) or caffeine placebo on 4 h night-time driving performance in healthy male volunteers.

## Methods

The protocol for this trial and supporting CONSORT checklist are available as supporting information; see Checklist S1 and Protocol S1.

### Participants

24 young (20–25 years) and 24 middle-aged (40–50 years) healthy male volunteers (mean age 33.19±10.91) were recruited (January 2010–June 2011) via advertisements (at Universities, organizations or hospitals in Bordeaux) or internet announcements. All volunteers had a BMI between 18 and 25 kg/m^2^ and were free from medical, psychiatric and sleep disorders as determined by questionnaires (SCL 90 R [12], Basic Nordic Sleep Questionnaire [13], Epworth Sleepiness Scale [14], sleep recordings (actigraphy (Actiwatch®, Cambridge Neurotechnology, UK)), nocturnal polygraphic recording (Embletta Gold®, EMBLA, NL)) and clinical interview with a medical doctor. They should maintain their normal and preferred sleep schedule (bedtime midnight ±2 hr and waketime 8:00 AM ±2 hr). They were not taking any treatment interfering with sleep, alertness or the circadian system and were moderate consumers of coffee (2–3 cups a day). They had had their driver's license for at least 3 years and drove between 10 000 and 20 000 km per year. Night workers and professional drivers were not included. Participants gave their written and informed consent to the study, which was approved by the local ethics committee (committee for the protection of persons participating in biomedical research, CPP Bordeaux A). In the consent, as French legislation requires, the objectives of the study are clearly exposed. The study was declared as a Clinical trial (ClinicalTrials.gov identifier: NCT1070004).

### Study design and randomisation

**Figure 1:**
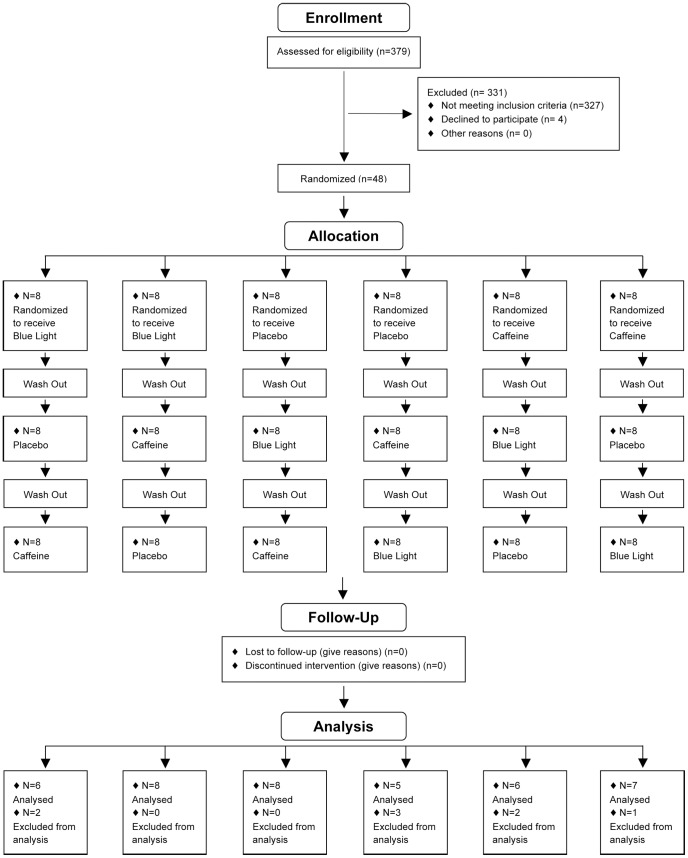
Participant flow: The numbers of participants who were randomly assigned, received intended treatment and were analyzed for the primary outcome.

**Figure 2:**
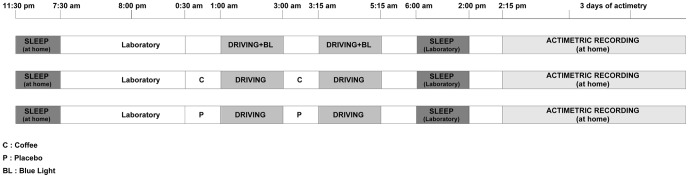
Protocol Design: sleep-driving period for the three substance conditions (placebo, coffee and blue light).

This was a randomized placebo-controlled crossover study with 2 double-blind conditions: coffee-placebo intake and a continuous blue light exposure condition (Figure 1). For each driving session, participants drove on the same motorway for 4 hours with a 15-minute break in the middle of the session. They randomly received either continuous blue light exposure during driving or 2*200 mg of coffee or placebo of coffee before driving and during the break. They were informed that the study was conducted in order to test the effects of countermeasures (coffee and blue light) on driving ability, that they will receive an undisclosed placebo and that we awaited countermeasures to improve the driving performance. There was at least 1 week between each condition and the order of sessions was randomly attributed to each participant. The order of administration was determined using computer-generated random numbers. The randomization is based on a randomized Latin square to form six possible combinations from the three treatments, and the three groups were balanced (2 subjects/group). Randomization and implementation procedures were performed by a person from the clinical investigation unit not involved in the clinical trial. Participants and other research staff involved in the trial were blinded to the assignments (Placebo/coffee). No stimulant of any kind was allowed during the study except for the scheduled caffeine condition. After driving, participants returned to the laboratory to sleep during the daytime (Figure 2). Participants were instructed to maintain their usual-preferential sleep schedules (habitual sleep/wake timing ±30 min and sleep duration) verified by actimetric recordings 3 days prior each driving sessions. To verify the short-term effect of countermeasures on the quality/quantity of sleep and the regularity of the sleep wake cycle, activity was recorded by actigraphy for 3 days after each condition.

### Driving Sessions

The night-time driving session started at 1:00 AM and finished at 5:15 AM. All participants drove 400 km (250 miles) on the same 2-lane motorway for all conditions. After 2 hours of driving (200 km, 125 miles), subjects took a 15-minute break. Driving conditions were a straight motorway on weekdays with usually light traffic conditions and in fair weather. Subjects were instructed to maintain a constant speed (130 kph; 80 mph), to drive in the center of the lane, and not to cross the painted lines separating the lanes, except to overtake a slower vehicle. During the whole experiment, a professional driving instructor monitored the driving speed and was ready to take control of the car (which was equipped with dual controls) if needed. No verbal communication was allowed between the drivers and co-pilots unless a specific instruction had to be given (e.g., stop to the next rest area). If a participant could no longer drive during a session, the participant was driven back to the rest area.

### Coffee and Placebo

Coffee and placebo were prepared from single packs of the relevant instant coffee (normal or decaffeinated) provided by Nestlé (Nestlé, Noisiel, FR). Coffee contained 4.25% caffeine and placebo (decaffeinated coffee) contained less than 0.3% caffeine. Placebo and coffee were not distinguishable by taste or appearance. Each participant drank 125 ml of coffee (about half a cup of coffee, containing 200 mg of caffeine) or 125 ml of placebo (containing 15 mg of caffeine) 15 minutes before driving and at the beginning of the break.

### Blue Light Exposure

Blue light (goLITE BLU®, Philips, NL) was LED light sources with a spectral wavelength of 468 nm±8 nm). The light source (14× 14×2.5 cm) was placed in the middle of the dashboard at approximately a 25° horizontal angle of gaze and approximately 75 cm from the participant's eyes. The light device was used according to the manufacturer's recommendations in order to increase safety and avoid blue light hazard. Luminance at the eye level was in order of 20 lux with an intensity of 7.4 µw/cm2.

### Outcome Measures

The main driving ability criterion was the number of inappropriate line crossings (ILC). This measure was selected because epidemiologic findings have shown that 65% of sleep-related accidents occur after an ILC [15]. Several studies have also shown that impaired daytime alertness induces lateral deviations during driving [16], [17], [18] and that sleep-related accidents frequently occur with a single car driving off the road and hitting an obstacle with no reaction from the driver [15], [19]. We have also demonstrated that the number of ILCs is affected by sleep deprivation [20], [21] and improved by classic countermeasures to sleep loss [3]. ILC was identified by a Continental Automotiv® video system, which measures and registers the lateral position (cm) of the car (10 times/sec) from the right lateral lane marker of the road. The Continental Automotiv® video system was calibrated according to the lane characteristics. An ILC was recorded when the car crossed a right or left lateral lane marker, whatever the duration and the amplitude of the crossing. Exceptions were overtaking manoeuvres or some other necessary driving action. All ILC were confirmed manually by video-recording analysis. The scorer of video recordings was blind to the driving condition. Lateral Position was defined as being 0 when the car was in the center of the lane, with positive value to the right and negative values to the left.

Standard deviation of the lateral position of the car (SDLP in cm), derived of lateral position, indicates weaving of the car, used as another parameter to identify driving performance [22].

Sleep duration, quality and timing of 3 subsequent sleeps were determined by actigraphy (Actiwatch®, Cambridge Neurotechnology, UK). This device monitors body movements and allows calculation of nocturnal sleep episodes and nocturnal awakenings. Time in bed was also computed as the time difference between going to bed in the evening and getting up in the morning. Sleep efficiency was calculated as the ratio of time asleep to time in bed expressed as a percentage.

### Statistical Analysis

Driving performance and actimetric recordings were analyzed by mixed-model analysis using a composed symmetry structure to adjust for serial correlation across time.

For driving performances, the dependant variables were mean lateral position, mean cumulative number of ILC and mean standard deviation of the position of the car, while the predictive factors were age (young vs. middle-age), substance (placebo vs. coffee vs. blue light), and driving session (first vs. second). For actimetric recordings, the dependant variables were sleep efficiency, total sleep time and bedtime recorded after the driving sessions, while the predictive factors were age (young vs. middle-age), substance (placebo vs. coffee vs. blue light), and day (first vs. second vs. third). The subject was considered as a random intercept assumed to be constant across conditions. The fully saturated model was run and the final mixed model included all main effects and interaction terms. Fisher's LSD post-hoc comparisons were used when a significant difference was found. Moreover, a non parametric Wilcoxon test was used to examine the driving performance of participants complaining about dazzle under blue light and placebo. T-test was also used to compare the driving performance of the participants with and without the participants complaining about dazzle. The SPSS® statistical package (Version 18; Chicago, USA) was used for all analyses. Statisticians were blinded to the assignments (Blue light/placebo/coffee).

## Results

Eight data out of 288 (3%) were not included in the analysis. They concerned 5 drivers who did not manage to finish one or various second night-time driving sessions because they were too sleepy to drive. Three incomplete driving sessions were under placebo, 2 under coffee and 3 under continuous blue light exposure.

As some participants complained about dazzle during continuous blue light exposure and as the variability of driving performance (ILC) was high for this driving condition [Standard error (SE) for Blue light = 6.15, for Placebo = 5.12 and for Coffee = 2.72)], a hierarchic classification based on the Ward method was used to check whether all participants performed equally. The result showed that 8 participants (17%) exhibited a significantly higher number of ILC (102.38±13.22) than the others (14.58±2.22). The deterioration of driving performance of these 8 participants was higher under blue light session than under placebo (ILC, first driving session = 67±17 versus 37.5±16, p = 0.04 ; second driving session 138±10 versus 46±11 p = 0.01, for blue light and placebo respectively, wilcoxon non parametric test). Results from these 8 participants (3 young and 5 middle-aged) were thus removed. Furthermore, removing from analysis the eight participant who complained of dazzle did not modify the number of inappropriate line crossing under placebo condition (first driving session = 23±4 (n = 48) versus 20±4 (n = 40), p = 0.65 ; second driving session = 35±6 (n = 48) versus 33±6 (n = 40), p = 0.81, t-test).

### Driving performance

Mean lateral position of the car was 23.00 cm (95% confidence interval [CI], 22.31 to 30.98) (toward the right) with placebo, and closer to center of the lane with coffee (19.55; 95% CI, 16.36 to 25.01, P = 0.004) and continuous blue light exposure (20.94; 95% CI, 18.15 to 26.81,, P = 0.004 respectively; (F[2, 188] = 4.98, P = .008).

**Figure 3:**
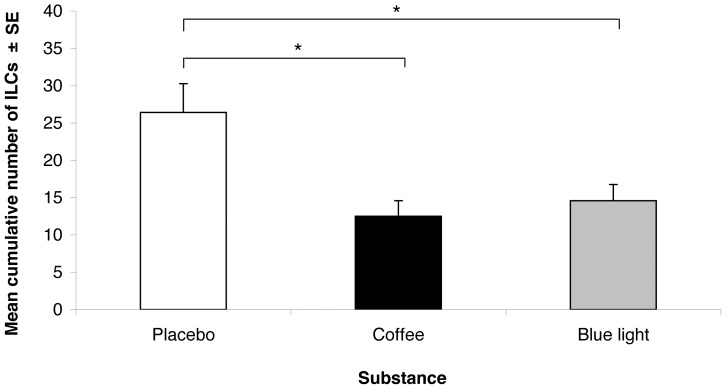
Driving Performance: Mean cumulative number of inappropriate line crossings in all substance conditions. * P<0.01.

Both countermeasures improved the driving performance (F[2, 91] = 6.63, P = .002) as the number of ILC was higher with placebo than with coffee (26.42; 95% CI, 19.90 to 33.71 vs 12.51; 95% CI, 5.86 to 19.66, P = .001) and continuous blue light exposure (26.42; 95% CI, 19.90 to 33.71 vs 14.58; 95% CI, 8.75 to 22.58, P = .003) (Figure 3). A significant effect of the moment of driving was also found (F[1, 104] = 11.47, P = .001), indicating a higher number of ILC during the 2^nd^ night-time driving session than during the 1st night-time driving session (21.32; 95% CI, 16.37 to 28.04 vs 14.59; 95% CI, 8.86 to 20.38, P = .001). No significant effect of age and no significant interaction were found.

Countermeasures and driving session had significant effects on the mean standard deviation of the position (F[2, 99] = 9.54, P = .001 and F[1, 95.76] = 20.07, P = .001, respectively). Indeed, the deviation was smaller with coffee than with placebo (24.74; 95% CI, 22.95 to 26.55 vs 28.56; 95% CI, 26.93 to 30.53, P = .001) and tended to be smaller with continuous blue light exposure (24.74; 95% CI, 22.95 to 26.55 vs 26.87; 95% CI, 25.22 to 28.83, P = .051). Moreover, a greater deviation was found during the second night-time driving session than during the first one (25.53; 95% CI, 26.51 to 29.75 vs 27.99,95% CI, 23.93 to 27.14, P = .001). No other significant effect or interaction was found.

### Timing, quantity and quality of 3 subsequent sleeps

The results indicated a significant effect of Age on sleep efficiency (F[1, 78] = 11.1, P = .001). Indeed, middle-aged subjects had higher sleep efficiency than young subjects (89.36; 95% CI, 87.59 to 91.14 vs 85.24; 95% CI, 83.52 to 86.95). No effect of Substance was found.

There was a significant effect of Day on Total Sleep Time (F[2, 252] = 10.17, P = .001), indicating a longer sleep time on the first day than on the third (455.85; 95% CI, 436.90 to 474.80 vs 421.62; 95% CI, 402.23 to 441.01, P = .01) or second ones (455.85; 95% CI, 436.90 to 474.80 vs 389.90; 95% CI, 379.91 to 417.90, P = .001). No effect of Substance was found.

Bedtime was influenced by Age and Day (F[1, 76] = 660.6, P = .001 and F[2, 230] = 3.91, P = .021, respectively). Middle-aged subjects went to bed earlier than young subjects (11.44 pm; 95% CI, 139.03 to 187.47 vs 1.25 am; 95% CI, 247.73 to 294.48, P<.001). Participants went to bed earlier on the first day than on the second one (0.16 am; 95% CI, 174.93 to 220.68 vs 0.56 am; 95% CI, 212.94 to 258.85, P = 0.006). No effect of Substance was found.

## Discussion

The main findings of this randomized controlled study were that continuous blue light exposure during nocturnal driving resulted in significantly reduced ILC and weaving compared with caffeine placebo, and that it was similar to caffeine (a countermeasure reference) in improving driving ability. Both countermeasures caused a driving closer to the center of the road. Continuous nocturnal blue light exposure was effective in short and long driving periods and throughout the night, even at the circadian trough. The alerting effect of blue light exposure was observed in both young and middle-aged drivers, even if age-related changes are known to reduce light transmission, particularly that of blue light [23]. Chellappa [24] has demonstrated that humans homozygous for the PER3 5/5 allele are particularly sensitive to blue-enriched light, so continuous blue light exposure during nocturnal driving could be proposed in first intention to these specific drivers.

Recently, we demonstrated the gradual standard deviation of lateral position increment during prolonged nocturnal motorway driving [25] and its relationship with alcohol-induced impairment [26]. Nocturnal driving impairment under placebo corresponds to a blood alcohol concentration (BAC) close to 0.10%. Nocturnal driving impairment under continuous blue light exposure and caffeine intake corresponds to a blood alcohol concentration (BAC) inferior to 0.08% (below the legal limits of United Kingdom and some US states).

Our results are consistent with those of a previous randomized trial of blue light or blue-enriched light on alertness and cognitive performance. That study demonstrated that nocturnal exposure to blue light was effective in enhancing cognitive performance on a sustained attention task [2], [10], [27], [28], but not on higher execute functions tasks [10]. In contrast with our results, blue-light exposure was not reported to increase driving simulator performance [27]. This inconsistency may be due to the lower blue light intensity used in the previous study (1 lux, 2 uw/cm2).

Using a sustained attention task, various studies showed a better tolerance to sleep deprivation with aging [29], [30], [31]. Blatter [32] showed that young and old adults exhibited similar sustained attention decrements during the night. The present study confirms our previous finding that nocturnal driving impairment is not affected by age [4].

On the one hand, occasional continuous nocturnal blue light exposure has no residual effect on quantity and timing of subsequent sleep. On the other hand, 17% of drivers experienced eye-related discomfort and/or visual problems. This discomfort greatly impaired the ability to maintain a stable lane position. Drivers should be informed about this side-effect and screening should be performed to identify blue light-intolerant drivers. These drivers should not use blue light as a countermeasure to fight nocturnal sleepiness. The complaints about dazzle made by some subjects could not be due to the high irradiance level used in our study. The irradiance level commonly used in studies demonstrating the beneficial effect of blue light on nocturnal alertness is higher [2], [11]. To increase tolerance, we suggest testing better placement of the light source panel in the car (e.g. above the driver's head) on nocturnal driving performance. Even if the light device used in this study has been tested for ocular safety [33], the potential risks of retinal damage due to blue light hazard [34] should not be forgotten particularly with regard to long-term use.

A limitation of our study is that the number of participants was small and the population limited to men owing to the effects of menstrual cycle phase on cognitive performance during sleep deprivation [35]. Future studies are needed to investigate the effect in sleep-deprived women. Furthermore, this study tested the effect of occasional continuous nocturnal blue light exposure. Future studies should investigate the effect of continuous nocturnal blue light exposure used repeatedly.

In conclusion, we demonstrated that, provided it does not dazzle drivers, continuous nocturnal blue light exposure could be used as an in-car countermeasure to fight nocturnal sleepiness at the wheel in both young and middle-aged drivers. Used occasionally, it does not affect subsequent sleep. Despite these findings, this clinical trial is a small one. More studies are needed to determine the reproducibility of data and to verify if it can be generalized to women.

## Supporting Information

Checklist S1 **CONSORT Checklist.**
(DOC)

Protocol S1 **Trial Protocol.**
(DOC)
