# Supplementary material for: In-Car Nocturnal Blue Light Exposure Improves Motorway Driving: A Randomized Controlled Trial
Source: PLoS One. 2012 Oct 19;7(10):e46750. doi: 10.1371/journal.pone.0046750 (PMC3477137; doi:10.1371/journal.pone.0046750)
Supplement: Protocol S1 — Trial Protocol. (DOC) [file pone.0046750.s002.doc]

**The effects of blue light versus coffee and placebo on night-time highway driving: a study of inter-individual differences**

**Background**

Sleep deprivation induces degradation of night-time driving ability via sleepiness. Because of conflicts between physiological needs and social or professional activities, it is necessary to develop affordable countermeasure to sleepiness. In real-life driving studies, nap and coffee are efficient countermeasures of sleepiness at the wheel. However the effect of caffeine is quick but brief and varies between individuals. There is a need for more knowledge in order to know what to recommend to drivers. Exposure to 460-nm monochromatic light (blue light) decreases subjective sleepiness and improves performances. One objective of this project is to investigate whether blue light exposure during driving would be useful in a real driving situation when sleepiness becomes acute. Owing to the fact that our knowledge of the effects of exercise on driving is very sparse and to the absolutely need to standardize the bouts of exercise that will be applied to the subjects. One objective of the present study will be to investigate in a simulator study the effects of a bout of moderate exercise on participants driving ability when sleepiness becomes acute. Nocturnal neurobehavioral performance varies widely between individuals and only certain subjects seem significantly affected by sleep loss. It is of interest to find biological markers for sleep drive to identify vulnerable drivers to sleep deprivation or to identify responders to sleepiness countermeasures (i.e., coffee and blue light). One objective of this study is to determine individual differences (genetic, hormonal and cognitive) in the impairment of driving skills induced by sleep loss and in the efficiency of countermeasures (blue light and coffee).

**Study objectives**

Primary objective

To compare the effects of continuous blue light exposure during driving with those of coffee (2*200 mg of caffeine) and coffee placebo on 4h night-time driving performance in young (20-25 years) and middle-aged (40-50 years) healthy volunteers

Secondary objectives

To determine the effect of age in the effectiveness of countermeasures (blue light and coffee)

To determine individual differences (cognitive, genetic and hormonal) in the impairment of neurobehavioral functions from sleep loss and in the effectiveness of countermeasures (blue light and coffee)

To determine the effects of blue light on quality and quantity of sleep after night-time driving in young and middle-aged healthy volunteers

**Study design**

Experimental design

This is an interventional study: Double-blind (except for blue-light), Randomized, Crossover, Comparative versus referent countermeasure of sleepiness: coffee (2*200 mg of caffeine) and placebo.

The study is divided into the following periods:

1. **selection period** without treatment between selection and inclusion visits to check the absence of exclusion criteria with questionnaires, polygraphic recording (respiratory sleep disorders and periodic leg movements) and actimetry (sleep efficiency, total sleep time)
2. **acute treatment period**, each volunteer will be randomly allocated to successively and randomly receive either continuous blue light exposure or 2*200 mg of caffeine or placebo of caffeine during night driving with at least 1 week between treatment.

Experimental protocol

SELECTION

Participant will complete the French version of the Basic Nordic Sleep Questionnaire (BNSQ), the self-report symptoms inventory SCL-90R, the chronotype questionnaire of Horne and Ostberg, the Epworth sleepiness scale. They will to have 7 consecutive days monitoring using actimeters and 1 night using polygraph.

INCLUSION

Participants will be screened for eligibility criteria. They will sign informed consent, perform cognitive tests, complete the caffeine sensitivity questionnaire. Blood samples will be collected.

DRIVING SESSION

Each subject, will come at the laboratory at 9:30 PM, and will kept awake until the time of the experimental session.

For each driving session (Blue light, caffeine and placebo of caffeine), all participants will drive 400 km (250 miles) on the same 2-lane highway for 4 hours. After 2 hours of driving (200 km, 125 miles) subjects will take a 15-minutes break. The night-time driving session will start at 1:00 AM and Stop at 5:15 AM. At the beginning, middle and end of the driving, participants will be asked to rate their instantaneous fatigue and sleepiness. After driving, sleep recuperation will be recorded in laboratory during a minimum of 6h. Saliva will be collected before and after the driving session and after sleep recuperation.

The subjects were instructed to maintain a regular sleep-wake schedule and were monitored by actimetry during the 3 days preceding each experimental session and during the 3 days after each experimental session.

**
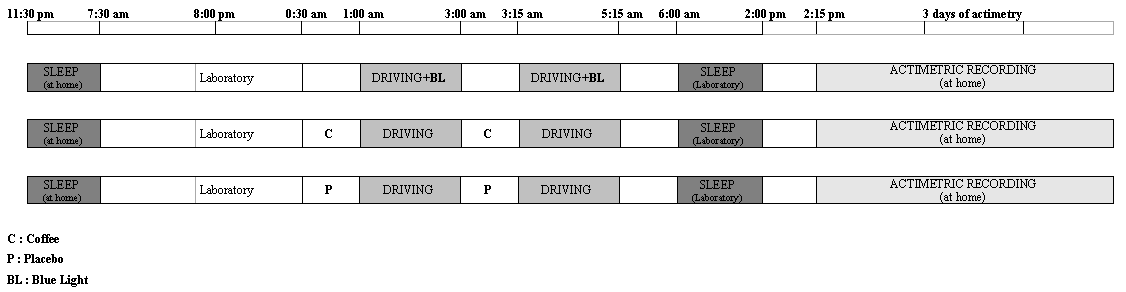
**

C: Coffee

P: Placebo

BL: Blue Light

**Selection of participants**

Inclusion criteria

- Healthy volunteers
- Aged between 20-25 years or 40-50 years
- Male
- BMI between 18 and 27
- Moderate caffeine drinkers (2-3 cups by day)
- Non professional drivers had their driving license for at least 2 years and drove between 10000 and 20000 km per year
- Showing a morning or evening chronotype (Horne questionnaire score >58 or < 42 for young subjects and Horne questionnaire score >64 or < 53 for middle-aged subjects)

The patient’s informed consent must be obtained at the inclusion.

Exclusion criteria

- Evidence of psychopathology (self-reports symptom inventory SCL-90R score >59 on the general symptomatic index and the following symptom dimensions: depression, anxiety, paranoid ideation and psychoticism)
- Evidence of sleep disorders (Items of Basic Nordic Sleep Questionnaire >3),
- Evidence of excessive daytime sleepiness (Epworth sleepiness scale score >9)
- Hypopnea-apnea index ≥ 5 (assessed by polygraphy)
- Periodic leg movement index ≥ 15 (assessed by polygraphy)
- Sleep efficiency < 85 % (assessed by actimetry during 7 consecutive days)
- Shift-workers and jet-lag the month before their inclusion in the study
- Severe controlled or uncontrolled diseases (psychiatric disorders, neurological disorders, sleep disorders, hepatic or renal failure, unstabilized diabetes, neoplasic disorders, cardiovascular disorders, pulmonary disorders, digestive disorders,…)

**Treatment**

Blue light

The subjects will be continuously exposed of 460-480 nanometers monochromatic light for 4 hours during nighttime driving.

The Apollo goLite M2 with Long Lasting Eye Safe LEDs technology will be fixed in the car. This product, CE Safety Tested, is distributed by Respironics-Philips and used in many countries. This device produces no UV or near-UV light, and passes ocular safety testing as well as all government and industrial ocular standards

Coffee

Each participant will drink 125 ml of coffee (about half a cup of coffee, containing 200 mg of caffeine) or 125 ml of placebo (containing 15 mg of caffeine) 30 minutes before the nighttime driving session and after 2 hours of driving.

Coffee and placebo will be prepared from single packs of the relevant instant coffee (normal or decaffeinated) provided by Nestlé. Coffee contains 4.25% caf­feine and placebo (decaffeinated coffee) contains less than 0.3% caffeine. Placebo and coffee will not distinguishable by taste or appearance.

**Criteria of assessment**

Primary efficacy criterion

- Number of inappropriate line crossings (ILC) identified from the video recordings.

Secondary efficacy criteria

- Standard deviation of the position of the car
- Self-rated sleepiness during driving
- Self-rated fatigue during driving
- Sleep latency during the subsequent sleep
- Sleep efficiency during the subsequent sleep
- Time course of EEG slow wave activity during the subsequent sleep

Secondary criteria

- Chronotype
- Caffeine sensitivity
- Habitual sleep patterns
- PER3 polymorphism
- COMT polymorphism
- ADORA2A polymorphism
- Reaction time and percentage of errors at cognitive tests
- Saliva cortisol concentration before and after the driving session and after the sleep recovery.
- Saliva amylase concentration before and after the driving session and after the sleep recovery.

**Clinical assessment methods**

EFFICACY

Driving session

Driving conditions will be a straight highway on weekdays with usually light traffic conditions, in fair weather. All drivers will be exposed to the same or very similar conditions. During a training session, participants will be instructed to maintain a constant speed (130 kph [80 mph]), to drive in the center of the lane, and not to cross the painted lines separating the lanes except to pass a slower vehicle.

During the whole experiment, a professional driving instruc­tor will monitor the driving speed. He will be ready to take control of the car (equipped with dual controls) if needed. If a participant could no longer drive during a session, he will drive back to the rest area. The car used for the experiment will be equipped with a video system that calculates the driving ability.

The main outcome measure will be the number of inappropriate line crossings (ILC) identified from the video recordings. This measure was selected because epidemiologic findings have shown that 65% of sleep-related accidents occur after an ILC (Sagberg 1999). Several studies have also shown that impaired daytime alertness induces lateral devia­tions during driving (O'Hanlon and Volkerts 1986; Ramaekers and O'Hanlon 1994; O'Hanlon, Vermeeren et al. 1995) and that sleep-related accidents frequently occur with a single car driving off the road and hitting an obstacle with no reaction from the driver (Pack, Pack et al. 1995; Sagberg 1999). We have also demonstrated that the number of ILCs is affected by sleep deprivation (Philip, Sagaspe et al. 2005; Philip, Sagaspe et al. 2005) and improved by classic countermeasures to sleep loss (Philip, Taillard et al. 2006).

An ILC is recorded when the car cross a lateral highway lane marker, as evidenced by video-recording analysis. Exceptions are overtaking manoeuvres or some other necessary driving action, as recorded by the driving instructor. Driving-instructor and video-recording timelines were synchronized at the beginning of each ses­sion. Deviations related to traffic interference will be excluded so as to concentrate on line crossings related to driver status.

The second outcome measure will be the standard deviation of lateral position. It is reasonable to assume that SDLP represents overall highway driving ability since it encompasses several levels of information processing which are combined to an integrated driving model (Ranney 1994). For example, basic vehicle control, such as road tracking, is required involving automatic or effortless performances. Further, negotiation of common driving situations, such as curves, intersections, and gap acceptance, requires controlled processing, which is more effortful. Since SDLP increment ultimately results in lane crossing into the adjacent traffic lane, SDLP can be regarded as an index of driving safety. Furthermore, this test is performed during normal traffic; hence, the on-the-road driving test is a close representation of normal driving.

Subjective sleepiness

Participants will be asked to rate their sleepiness on the Karolinska Sleepiness Scale (a 9-point scale from 1 = “extremely alert” to 9 = “very sleepy, great effort to keep alert, fighting sleep”).

Subjective fatigue

Participants will be asked to rate their instantaneous fatigue (“de­scribe how fatigued you are now” on a 100-mm visual analogue scale from 0 “not at all tired” to 100 “very tired”).

Polysomnography

Three electroencephalograms (F3/A2, C3/A2, O1/A2), 1 electromyogram, 2 electrooculograms, and 1 electrocardiogram will be recorded dur­ing the night after driving.

Signals will be digitized at a sampling rate of 256 Hz and filtered with a digital filter at a cutoff frequency of 35 Hz.

Data will be manually analyzed by an experienced sleep technician in 30-second epochs according to Rechtschaffen and Kales’ recommendations (Rechtschaffen and Kales 1968). Non-rapid eye movement (NREM) sleep (stages 2, 3, and 4) during sleep will be retained for spectral analysis. Electroencephalographic signals of C3/A2 derivation will be subjected to analysis by fast Fourier transforma­tion. Power spectra will compute for consecutive 2-second epochs, providing a frequency resolution of 0.5 Hz. The delta frequency band (0.5-4.5 Hz) will be exploited.

SUBJECTS TYPING

Genomic (PER3, COMT, ADA, ADORA2A) DNA

Genomic DNA will be extracted from 2*5ml blood sample (tube with EDTA solution).

Saliva cortisol and amylase

Saliva will be directly collected from mouth to tube. Samples will be stored at -18°c before analysis. Saliva cortisol and amylase concentrations will be determined by RIA.

Caffeine sensitivity assessment

Subject reporting nocturnal sleep disturbances after caffeine intake in the afternoon will be considered as caffeine sensitive and subject reporting no problems sleeping after caffeine in the afternoon were considered as caffeine insensible.

Cognitive tests : Evaluation of attentional components

*- Tonic and phasic Alertness*: The examination includes a simple and a cued reaction time task with a visual test stimulus and an acoustic cue. The difference between simple and cued reaction time is a measure of phasic alertness. (Computerized test)

- *Vigilance and sustained attention*: There are four tasks with different stimuli (3 unimodal tasks: 1 acoustic, 2 visual; one bimodal task: visuo-acoustic) that assess sustained attention or vigilance. Each task can be run with high (sustained attention) or low event rate (vigilance) of critical stimuli. (Computerized test).

*- Divided attention*. A dual task is used. The visual task consists of crosses that appear in a random configuration in a 4 x 4 matrix. The subject has to detect whether the crosses form the corners of a square. The acoustical task includes a regular sequence of high and low beeps. The subject has to detect an irregularity in the sequence. (Computerized test).

- Selective attention (Zazzo’s cancellation task, short 8-line version). This test measures the ability to cross out as fast and as exactly as possible target signs among distracters on a sheet of white paper containing 8 lines of signs. In our study, we considered the time spent to complete all 4 lines and the omission errors and false alarms. (Pen-paper test)

Cognitive test : Evaluation of executive functions

Inhibition

- Go/No-Go: this test requires a subject to emit a simple motor response (Go) to one cue while inhibiting the response in the presence of another cue (No Go). There are twice more Go signals than No-Go signals. Reaction time and percentage of error will be measured.

- Incompatibility: during incompatibility task , the tendency of interferences will be tested by a Stimulus-Responses incompatibility. Subjects were instructed to squeeze as fast as possible in response to a left- or right-pointing arrow presented on a computer screen. The arrow determined whether the response was to be compatible (e.g., right arrow with right hand squeeze) or incompatible (right arrow with left hand squeeze). Reaction time and percentage of error will be measured in the two conditions.

Cognitive speed

- The Trail Making Test (TMT) R. Reitan, Trail Making Test: manual for administration and scoring, Reitan Neuropsychology Laboratory, Tuscon, AZ (1992) is used to assess cognitive speed, visual motor tracking, divided attention, and mental flexibility. It consists of two parts. In Part A, the participant has to connect numbers from 1 to 25 in the correct order as fast as possible (cognitive speed and visual motor tracking). (Pen and paper test). The score was total duration in seconds and the number of correct moves.

- The Digit Symbol subset of the WAIS-R is used to measure coding ability. This test is a performance measure which requires the subject to code nine simple symbols matched with nine numerals. The subject is given 90 seconds to code all items on the test. (Pen and paper test)

Flexibility

- TMT-B : numbers from 1 to 13 and letters from A to M must be connected in alternating fashion, beginning at 1-A and ending at M-13. Total score is given by time spent to complete each part (Pen and paper test)

- TEA Flexibility : Two stimuli (letter and digit) are displayed at a time. Subjects will be instructed to push on right or left key in according to the target. (Computerized test)

Working Memory

Updating memory task. Subjects must recall digit number list.

SELECTION

Actigraphy

Actimeters will be used to quantify our volunteers’ sleep duration and quality. This device monitors body movements and allows calculation of mean nocturnal sleep episodes and of nocturnal awakenings. Time in bed will be also computed as the time difference between going to bed in the evening and getting up in the morning. Sleep efficiency will be calculated as the ratio of time asleep to the time in bed, in percentage. To rule out any sleep-wake schedule disorders each subject will be monitored for 7 days before being included in the study. Subjects will be included if they have a mean sleep efficiency of at least 85% during the 7 days of recordings.

Polygraphy

A nocturnal polygraphy (nasal flow, oxygen saturation, two respiratory effort belts, snore, position, leg movement) will be performed to eliminate subjects suffering from sleep disorders. Subjects presenting an AHI > 5 for the 20-25 years old and > 10 for the 40-50 years old or a PLM index >15 will be excluded of study.

Habitual sleep patterns

The evaluation of usual sleep schedules, sleep quality, sleep needs and sleep hygiene will be assessed by the Basic Nordic sleep questionnaire. This 22-items questionnaire evaluate the quality of sleep over the previous 3 months as measured by a frequency scale ranging from 0 (never or less than once a month) to 5 (almost everyday or everyday) (Partinen and Gislason 1995).

Chronotype assessment

The chronotype will be assessed by Horne and Ostberg questionnaire (Horne and Ostberg 1976). This questionnaire contains 19 items about individual rising and bedtimes preferred time of physical and mental performances and alertness after rising and before going to bed. It mainly use 4-choices items.

Sleepiness

Chronic daytime sleepiness will be assessed by the Epworth Sleepiness Scale wich rates the tendency to fall asleep in 8 different situations in daily life.

Self-reports symptom inventory SCL-90R

The evidence of psychopathology will be assessed by the Symptom Checklist 90 (SCL-90). This questionnaire is a psychiatric self-report inventory. The 90 items in the questionnaire are scored on a five-point Likert scale, indicating the rate of occurrence of the symptom during the time reference. It is intended to measure symptom intensity on nine different subscales. It has been shown to have a good reliability as its internal consistency is high.

**Statistical considerations**

Determination of sample size

The sample size calculation (G power software) was done in order to allow demonstrating a slight difference between the 2 countermeasures (coffee and blue light) with a power of 90 % and a type-I error =5% approximately 48 subjects have to be included in the study:

Effect size f = 0.3

α _err prob_ = 0.05

Power (1-β _err prob_) = 0.9

Total sample size: 48 **(24 young and 24 middle-aged)**

**Analyses of variables**

Analysis of primary efficacy variable

The results will be analyzed by using negative binomial regres­sion in Stata, version 8.0 (Stata Corp., College Station, Texas), using number of ILC per participant as dependent variables and treatments (Blue light, coffee, or placebo), age or type as determi­nants clustered on participants. We reported comparisons of ILC between treatments as incidence rate ratios (IRR) with 95% confi­dence intervals (CI). Where binomial negative regression are not possible because there are no line crossing, the Wilcoxon non­parametric test will be used.

Analysis of secondary efficacy variables

The standard deviation of the position of the car will be compared by a two-way analysis of variance (ANOVA) (age*treatment, genotype*treatment, hormonal type *treatment, cognitive type*treatment, …).

Subjective sleepiness or subjective fatigue will be compared by a three-way analysis of variance for repeated measurements (rANOVA) (time*age*treatment, time*genotype*treatment, time*hormonal type*treatment, time*cognitive type*treatment, …)

Sleep latency, sleep efficiency or sleep EEG power density will be compared by a three-way analysis of variance for repeated measurements (rANOVA) (time*age*treatment, time*genotype*treatment, time*hormonal type*treatment, time*cognitive type*treatment, …).

**Expected results**

- Improvement of nocturnal driving ability with countermeasures to sleepiness (coffee and continue blue light exposure) versus placebo in young and middle-aged healthy volunteers.

- Same beneficial effect of continue blue light exposure versus coffee on nocturnal driving ability in young and middle-aged healthy volunteers.

- Characterization of biological (genetic, cognitive or hormonal) markers explaining inter-individual variability in nocturnal driving impairment and effectiveness of countermeasures to sleepiness.

- Modifications on quality and quantity of sleep after the blue light exposure during nighttime driving in young and middle-aged healthy volunteers.
